# *N*,*N*,*N*′,*N*′-Tetra­ethyl-*N*′′-(2-fluoro­benzo­yl)phospho­ric triamide

**DOI:** 10.1107/S1600536811036944

**Published:** 2011-09-14

**Authors:** Atekeh Tarahhomi, Mehrdad Pourayoubi, Arnold L. Rheingold, James A. Golen

**Affiliations:** a Department of Chemistry, Ferdowsi University of Mashhad, Mashhad 91779, Iran; b Department of Chemistry, University of California, San Diego, 9500 Gilman Drive, La Jolla, CA 92093, USA

## Abstract

In the title compound, C_15_H_25_FN_3_O_2_P, the phosphoryl group is in an *anti* and *syn* orientation to the C=O and N—H groups, respectively. The P atom is in a distorted tetra­hedral environment. One of the ethyl groups is disordered over two sets of sites with refined occupancies of 0.755 (6) and 0.245 (6). In addition, the F atom was refined as disordered with occupancies fixed at 0.9 and 0.1. This disorder corresponds to a rotation of approximately 180° of the fluoro­benzene ring about its connecting C—C bond. In the crystal, pairs of inter­molecular N—H⋯O(=P) hydrogen bonds form centrosymmetric dimers.

## Related literature

For background to phospho­ric triamide compounds containing a C(=O)NHP(=O) skeleton, see: Pourayoubi *et al.* (2011*a* ▶,*b* ▶); Tarahhomi *et al.* (2011 ▶). For the synthesis of the starting material 2-F—C_6_H_4_C(=O)NHP(=O)Cl_2_, see: Pourayoubi *et al.* (2011*a* ▶). For hydrogen-bond motifs, see: Bernstein *et al.* (1995 ▶).
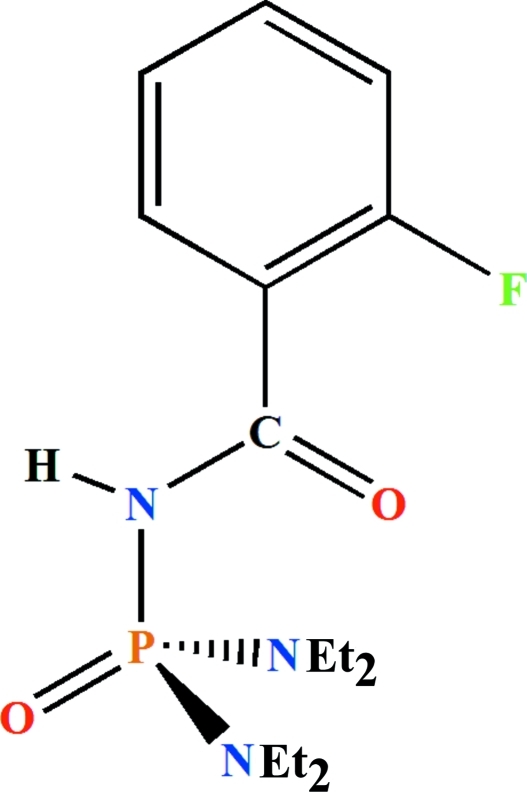


## Experimental

### 

#### Crystal data

C_15_H_25_FN_3_O_2_P
*M* *_r_* = 329.35
Monoclinic, 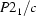

*a* = 10.9296 (9) Å
*b* = 12.2221 (10) Å
*c* = 12.3423 (10) Å
β = 91.443 (1)°
*V* = 1648.2 (2) Å^3^
*Z* = 4
Mo *K*α radiation
μ = 0.19 mm^−1^
*T* = 100 K
0.45 × 0.40 × 0.35 mm

#### Data collection

Bruker APEXII CCD diffractometer
Absorption correction: multi-scan (*SADABS*; Bruker, 2005 ▶) *T*_min_ = 0.920, *T*_max_ = 0.937
11336 measured reflections
3741 independent reflections
3056 reflections with *I* > 2σ(*I*)
*R*_int_ = 0.051

#### Refinement

*R*[*F* ^2^ > 2σ(*F* ^2^)] = 0.047
*wR*(*F* ^2^) = 0.122
*S* = 1.08
3741 reflections
236 parameters
6 restraints
H atoms treated by a mixture of independent and constrained refinement
Δρ_max_ = 0.55 e Å^−3^
Δρ_min_ = −0.42 e Å^−3^

### 

Data collection: *APEX2* (Bruker, 2005 ▶); cell refinement: *SAINT* (Bruker, 2005 ▶); data reduction: *SAINT*; program(s) used to solve structure: *SHELXS97* (Sheldrick, 2008 ▶); program(s) used to refine structure: *SHELXL97* (Sheldrick, 2008 ▶); molecular graphics: *SHELXTL* (Sheldrick, 2008 ▶); software used to prepare material for publication: *SHELXTL* and *enCIFer* (Allen *et al.*, 2004 ▶).

## Supplementary Material

Crystal structure: contains datablock(s) I, global. DOI: 10.1107/S1600536811036944/lh5308sup1.cif

Structure factors: contains datablock(s) I. DOI: 10.1107/S1600536811036944/lh5308Isup2.hkl

Additional supplementary materials:  crystallographic information; 3D view; checkCIF report

## Figures and Tables

**Table 1:** Hydrogen-bond geometry (Å, °)

*D*—H⋯*A*	*D*—H	H⋯*A*	*D*⋯*A*	*D*—H⋯*A*
N1—H1*N*⋯O2^i^	0.86 (2)	1.93 (2)	2.7714 (19)	167 (2)

Symmetry code: (i) 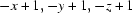.

**Fig. 1.**
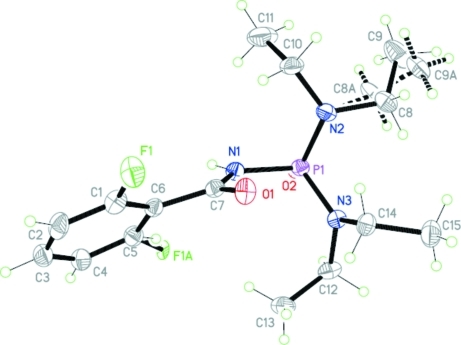
The molecular structure of the title compound. Displacement ellipsoids are given at 50% probability level and H atoms are drawn as small spheres of arbitrary radii. Disordered atoms are labeled with the suffix 'A'.

## supplementary crystallographic information

### Comment

Following our previous work on phosphoric triamides containing an XC(O)NHP(O) moiety, *X* = 2-F—C_6_H_4_ (Pourayoubi *et al.*, 2011*a*), 4-F—C_6_H_4_ (Tarahhomi *et al.*, 2011) and 2,6-F_2_—C_6_H_3_ (Pourayoubi *et al.*, 2011*b*), herein, we report the synthesis and crystal structure of the title compound, P(O)[2-F—C_6_H_4_C(O)NH][N(C_2_H_5_)_2_]_2_ (Fig. 1).

One of the —CH_2_CH_3_ groups in the diethylamido substituent N2 is disordered over two sets of sites with refined occupancies of 0.755 (6) and 0.245 (6). The fluorine atom of the aromatic ring is disordered over two sets of sites with occupancies of 0.9 and 0.1.

In the C(═O)NHP(═O) skeleton of the title phosphoric triamide, the phosphoryl group adopts the *anti* orientation with respect to the carbonyl group; whereas it is in a *syn* position relative to the N—H unit. The tetrahedral environment at the P atom is distorted as has been noted for the other phosphoric triamides. The P═O, C═O and P—N bond lengths are within the expected values (Pourayoubi *et al.* (2011*b*) and Tarahhomi *et al.* (2011)).

In the crystal, pairs of intermolecular N—H···O(P) hydrogen bonds form a hydrogen-bonded dimer with *R*_2_^2^(8) graph-set notation (Bernstein *et al.*, 1995).

### Experimental

2-F—C_6_H_4_C(O)NHP(O)Cl_2_ was prepared according to the literature method reported by Pourayoubi *et al.* (2011*a*).

The title compound was synthesized from the reaction of 0.40 g (1.56 mmol) 2-F—C_6_H_4_C(O)NHP(O)Cl_2_ with 0.456 g (6.24 mmol) of diethyl amine in dry chloroform (30 ml). The amine was added dropwise to a solution of 2-F—C_6_H_4_C(O)NHP(O)Cl_2_ at 273 K, with continuous stirring. After 17 h, the solvent was evaporated; the obtained solid was washed with distilled water and dissolved in a mixture of CH_3_OH/DMF [4:1 (*v*/*v*)]. Single crystals were obtained from this solution after a few days at room temperature. IR (KBr, ν, cm^-1^): 3061 (NH), 2970, 2886, 1675 (C═O), 1457, 1196, 1048, 946, 766.

### Refinement

Atoms C8 and C9 are disordered over two sets of sites with refined occupancies of 0.755 (6) and 0.245 (6). The fluorine atom is disordered over two positions and refined with the occupancy F1 and F1A fixed at 0.9 and 0.1 and the corresponding hydrogen atoms H1A and H5A were also treated as disordered. Hydrogen H1N was found in a Fourier difference map and refined with a N1—H1N distance was set at 0.87 (0.02) Å and allowed to refine with *U*_iso_ at 1.2 of parent N atom. All other hydrogen atoms were placed in calculated positions with C—H distances for CH_2_ of 0.99 Å, CH_3_ of 0.98 Å and C(Ar)H of 0.95 Å and with *U*_iso_ of 1.20 (or 1.5 for methyl H atoms) that of the parent C atom.

### Crystal data

**Table:**

C_15_H_25_FN_3_O_2_P	*F*(000) = 704
*M**_r_* = 329.35	*D*_x_ = 1.327 Mg m^−^^3^
Monoclinic, *P*2_1_/*c*	Mo *K*α radiation, λ = 0.71073 Å
Hall symbol: -P 2ybc	Cell parameters from 6938 reflections
*a* = 10.9296 (9) Å	θ = 2.5–27.9°
*b* = 12.2221 (10) Å	µ = 0.19 mm^−^^1^
*c* = 12.3423 (10) Å	*T* = 100 K
β = 91.443 (1)°	Block, colourless
*V* = 1648.2 (2) Å^3^	0.45 × 0.40 × 0.35 mm
*Z* = 4	

### Data collection

**Table:**

Bruker APEXII CCD diffractometer	3741 independent reflections
Radiation source: fine-focus sealed tube	3056 reflections with *I* > 2σ(*I*)
graphite	*R*_int_ = 0.051
φ and ω scans	θ_max_ = 28.0°, θ_min_ = 1.9°
Absorption correction: multi-scan (*SADABS*; Bruker, 2005)	*h* = −14→7
*T*_min_ = 0.920, *T*_max_ = 0.937	*k* = −16→14
11336 measured reflections	*l* = −15→16

### Refinement

**Table:**

Refinement on *F*^2^	Primary atom site location: structure-invariant direct methods
Least-squares matrix: full	Secondary atom site location: difference Fourier map
*R*[*F*^2^ > 2σ(*F*^2^)] = 0.047	Hydrogen site location: inferred from neighbouring sites
*wR*(*F*^2^) = 0.122	H atoms treated by a mixture of independent and constrained refinement
*S* = 1.08	*w* = 1/[σ^2^(*F*_o_^2^) + (0.0487*P*)^2^ + 0.7763*P*] where *P* = (*F*_o_^2^ + 2*F*_c_^2^)/3
3741 reflections	(Δ/σ)_max_ < 0.001
236 parameters	Δρ_max_ = 0.55 e Å^−^^3^
6 restraints	Δρ_min_ = −0.42 e Å^−^^3^

### Special details

**Table:**

Geometry. All e.s.d.'s (except the e.s.d. in the dihedral angle between two l.s. planes) are estimated using the full covariance matrix. The cell e.s.d.'s are taken into account individually in the estimation of e.s.d.'s in distances, angles and torsion angles; correlations between e.s.d.'s in cell parameters are only used when they are defined by crystal symmetry. An approximate (isotropic) treatment of cell e.s.d.'s is used for estimating e.s.d.'s involving l.s. planes.
Refinement. Refinement of *F*^2^ against ALL reflections. The weighted *R*-factor *wR* and goodness of fit *S* are based on *F*^2^, conventional *R*-factors *R* are based on *F*, with *F* set to zero for negative *F*^2^. The threshold expression of *F*^2^ > σ(*F*^2^) is used only for calculating *R*-factors(gt) *etc*. and is not relevant to the choice of reflections for refinement. *R*-factors based on *F*^2^ are statistically about twice as large as those based on *F*, and *R*- factors based on ALL data will be even larger.

### Fractional atomic coordinates and isotropic or equivalent isotropic displacement parameters (Å^2^)

**Table:**

	*x*	*y*	*z*	*U*_iso_*/*U*_eq_	Occ. (<1)
P1	0.62104 (4)	0.64819 (4)	0.52994 (4)	0.01901 (14)	
F1	0.88953 (13)	0.41423 (11)	0.83105 (10)	0.0338 (3)	0.90
F1A	0.7449 (9)	0.3460 (7)	0.4748 (4)	0.019 (2)	0.10
O1	0.83305 (12)	0.57884 (11)	0.68306 (10)	0.0259 (3)	
O2	0.52742 (11)	0.62160 (11)	0.44464 (10)	0.0230 (3)	
N1	0.66071 (13)	0.52777 (13)	0.58794 (12)	0.0199 (3)	
H1N	0.6116 (17)	0.4741 (15)	0.5750 (16)	0.024*	
N2	0.57927 (14)	0.72748 (13)	0.62914 (12)	0.0260 (4)	
N3	0.73894 (13)	0.70810 (12)	0.47587 (11)	0.0201 (3)	
C1	0.86735 (17)	0.34892 (16)	0.74688 (15)	0.0254 (4)	
H1A	0.8811	0.3968	0.8066	0.030*	0.10
C2	0.91255 (17)	0.24343 (17)	0.75327 (17)	0.0303 (5)	
H2	0.9557	0.2194	0.8167	0.036*	
C3	0.89453 (18)	0.17367 (17)	0.66716 (17)	0.0300 (4)	
H3	0.9265	0.1014	0.6704	0.036*	
C4	0.82999 (18)	0.20843 (16)	0.57575 (16)	0.0289 (4)	
H4	0.8176	0.1603	0.5161	0.035*	
C5	0.78342 (17)	0.31405 (16)	0.57156 (15)	0.0243 (4)	
H5A	0.7374	0.3366	0.5092	0.029*	0.90
C6	0.80224 (15)	0.38782 (15)	0.65610 (14)	0.0200 (4)	
C7	0.76718 (16)	0.50656 (15)	0.64574 (13)	0.0203 (4)	
C8	0.5579 (3)	0.8451 (2)	0.6092 (2)	0.0279 (7)	0.755 (6)
H8A	0.5829	0.8629	0.5347	0.034*	0.755 (6)
H8B	0.6103	0.8880	0.6601	0.034*	0.755 (6)
C9	0.4260 (3)	0.8792 (3)	0.6222 (3)	0.0463 (10)	0.755 (6)
H9A	0.4164	0.9562	0.6016	0.069*	0.755 (6)
H9B	0.4033	0.8698	0.6980	0.069*	0.755 (6)
H9C	0.3728	0.8338	0.5756	0.069*	0.755 (6)
C8A	0.4916 (9)	0.8158 (7)	0.5807 (7)	0.032 (2)	0.245 (6)
H8AA	0.5086	0.8280	0.5032	0.038*	0.245 (6)
H8AB	0.4054	0.7922	0.5867	0.038*	0.245 (6)
C9A	0.5141 (8)	0.9196 (7)	0.6449 (7)	0.033 (3)	0.245 (6)
H9AA	0.4629	0.9785	0.6147	0.049*	0.245 (6)
H9AB	0.6005	0.9403	0.6409	0.049*	0.245 (6)
H9AC	0.4935	0.9073	0.7208	0.049*	0.245 (6)
C10	0.54246 (19)	0.68425 (18)	0.73515 (15)	0.0297 (4)	
H10A	0.5370	0.7459	0.7868	0.036*	
H10B	0.6071	0.6341	0.7629	0.036*	
C11	0.4206 (2)	0.6232 (2)	0.7324 (2)	0.0485 (6)	
H11A	0.3562	0.6713	0.7029	0.073*	
H11B	0.4002	0.6010	0.8061	0.073*	
H11C	0.4271	0.5582	0.6865	0.073*	
C12	0.78030 (17)	0.66718 (17)	0.37025 (15)	0.0260 (4)	
H12A	0.8211	0.7276	0.3318	0.031*	
H12B	0.7077	0.6450	0.3259	0.031*	
C13	0.86787 (19)	0.57083 (18)	0.37903 (18)	0.0358 (5)	
H13A	0.9365	0.5897	0.4281	0.054*	
H13B	0.8989	0.5536	0.3072	0.054*	
H13C	0.8247	0.5071	0.4074	0.054*	
C14	0.82939 (17)	0.77616 (15)	0.53521 (15)	0.0241 (4)	
H14A	0.9105	0.7403	0.5329	0.029*	
H14B	0.8062	0.7812	0.6121	0.029*	
C15	0.8391 (2)	0.89111 (17)	0.48869 (18)	0.0343 (5)	
H15A	0.8554	0.8867	0.4111	0.051*	
H15B	0.9060	0.9304	0.5259	0.051*	
H15C	0.7620	0.9303	0.4991	0.051*	

### Atomic displacement parameters (Å^2^)

**Table:**

	*U*^11^	*U*^22^	*U*^33^	*U*^12^	*U*^13^	*U*^23^
P1	0.0162 (2)	0.0221 (3)	0.0187 (2)	0.00183 (17)	0.00024 (16)	0.00116 (17)
F1	0.0485 (8)	0.0294 (7)	0.0229 (6)	0.0034 (6)	−0.0118 (6)	−0.0017 (5)
F1A	0.025 (5)	0.014 (5)	0.018 (5)	0.003 (4)	−0.003 (4)	0.003 (4)
O1	0.0268 (7)	0.0235 (7)	0.0271 (7)	−0.0039 (6)	−0.0082 (5)	0.0007 (5)
O2	0.0172 (6)	0.0280 (7)	0.0236 (6)	−0.0007 (5)	−0.0028 (5)	0.0052 (5)
N1	0.0158 (7)	0.0218 (8)	0.0220 (7)	−0.0009 (6)	−0.0011 (5)	0.0005 (6)
N2	0.0284 (8)	0.0253 (8)	0.0245 (8)	0.0068 (7)	0.0064 (6)	0.0005 (6)
N3	0.0192 (7)	0.0224 (8)	0.0186 (7)	−0.0011 (6)	−0.0006 (5)	−0.0006 (6)
C1	0.0255 (9)	0.0283 (10)	0.0221 (9)	−0.0044 (8)	−0.0043 (7)	0.0018 (7)
C2	0.0247 (9)	0.0319 (11)	0.0338 (11)	0.0001 (8)	−0.0069 (8)	0.0140 (9)
C3	0.0236 (10)	0.0250 (10)	0.0414 (12)	0.0022 (8)	0.0037 (8)	0.0071 (9)
C4	0.0309 (10)	0.0237 (10)	0.0323 (10)	−0.0007 (8)	0.0043 (8)	−0.0004 (8)
C5	0.0254 (9)	0.0253 (10)	0.0222 (9)	0.0001 (8)	−0.0007 (7)	0.0025 (7)
C6	0.0152 (8)	0.0223 (9)	0.0223 (9)	−0.0007 (7)	0.0006 (6)	0.0043 (7)
C7	0.0198 (8)	0.0241 (9)	0.0170 (8)	−0.0002 (7)	0.0011 (6)	0.0021 (7)
C8	0.0256 (16)	0.0286 (15)	0.0294 (15)	0.0069 (12)	−0.0018 (12)	−0.0034 (11)
C9	0.0295 (17)	0.0396 (19)	0.069 (2)	0.0107 (14)	−0.0061 (14)	−0.0157 (16)
C8A	0.036 (5)	0.033 (5)	0.026 (4)	0.020 (4)	−0.017 (4)	−0.011 (4)
C9A	0.038 (5)	0.029 (5)	0.031 (4)	0.013 (4)	−0.003 (4)	−0.007 (4)
C10	0.0339 (11)	0.0328 (11)	0.0229 (10)	−0.0025 (9)	0.0099 (8)	−0.0048 (8)
C11	0.0439 (14)	0.0531 (16)	0.0498 (14)	−0.0144 (12)	0.0241 (11)	−0.0154 (12)
C12	0.0223 (9)	0.0356 (11)	0.0203 (9)	−0.0060 (8)	0.0034 (7)	−0.0037 (8)
C13	0.0316 (11)	0.0344 (12)	0.0420 (12)	−0.0005 (9)	0.0155 (9)	−0.0062 (9)
C14	0.0246 (9)	0.0221 (9)	0.0255 (9)	−0.0045 (8)	−0.0025 (7)	−0.0002 (7)
C15	0.0424 (12)	0.0231 (10)	0.0375 (11)	−0.0042 (9)	0.0012 (9)	0.0017 (9)

### Geometric parameters (Å, °)

**Table:**

P1—O2	1.4855 (13)	C9—H9A	0.9800
P1—N2	1.6354 (16)	C9—H9B	0.9800
P1—N3	1.6387 (15)	C9—H9C	0.9800
P1—N1	1.6885 (16)	C8A—C9A	1.513 (11)
O1—C7	1.222 (2)	C8A—H8AA	0.9900
N1—C7	1.374 (2)	C8A—H8AB	0.9900
N1—H1N	0.860 (15)	C9A—H9AA	0.9800
N2—C8	1.476 (3)	C9A—H9AB	0.9800
N2—C10	1.476 (2)	C9A—H9AC	0.9800
N2—C8A	1.553 (7)	C10—C11	1.526 (3)
N3—C14	1.473 (2)	C10—H10A	0.9900
N3—C12	1.478 (2)	C10—H10B	0.9900
C1—C2	1.382 (3)	C11—H11A	0.9800
C1—C6	1.396 (2)	C11—H11B	0.9800
C1—H1A	0.9500	C11—H11C	0.9800
C2—C3	1.373 (3)	C12—C13	1.520 (3)
C2—H2	0.9500	C12—H12A	0.9900
C3—C4	1.382 (3)	C12—H12B	0.9900
C3—H3	0.9500	C13—H13A	0.9800
C4—C5	1.388 (3)	C13—H13B	0.9800
C4—H4	0.9500	C13—H13C	0.9800
C5—C6	1.390 (3)	C14—C15	1.522 (3)
C5—H5A	0.9500	C14—H14A	0.9900
C6—C7	1.506 (3)	C14—H14B	0.9900
C8—C9	1.514 (4)	C15—H15A	0.9800
C8—H8A	0.9900	C15—H15B	0.9800
C8—H8B	0.9900	C15—H15C	0.9800
			
O2—P1—N2	117.38 (8)	N2—C8A—H8AA	110.3
O2—P1—N3	110.11 (7)	C9A—C8A—H8AB	110.3
N2—P1—N3	106.10 (8)	N2—C8A—H8AB	110.3
O2—P1—N1	105.95 (8)	H8AA—C8A—H8AB	108.6
N2—P1—N1	105.82 (8)	C8A—C9A—H9AA	109.5
N3—P1—N1	111.47 (8)	C8A—C9A—H9AB	109.5
C7—N1—P1	126.13 (13)	H9AA—C9A—H9AB	109.5
C7—N1—H1N	118.0 (14)	C8A—C9A—H9AC	109.5
P1—N1—H1N	115.7 (14)	H9AA—C9A—H9AC	109.5
C8—N2—C10	116.79 (17)	H9AB—C9A—H9AC	109.5
C8—N2—C8A	33.4 (4)	N2—C10—C11	114.35 (18)
C10—N2—C8A	114.2 (4)	N2—C10—H10A	108.7
C8—N2—P1	119.88 (15)	C11—C10—H10A	108.7
C10—N2—P1	122.61 (14)	N2—C10—H10B	108.7
C8A—N2—P1	107.7 (3)	C11—C10—H10B	108.7
C14—N3—C12	114.42 (14)	H10A—C10—H10B	107.6
C14—N3—P1	125.08 (12)	C10—C11—H11A	109.5
C12—N3—P1	117.98 (12)	C10—C11—H11B	109.5
C2—C1—C6	122.54 (18)	H11A—C11—H11B	109.5
C2—C1—H1A	118.7	C10—C11—H11C	109.5
C6—C1—H1A	118.7	H11A—C11—H11C	109.5
C3—C2—C1	119.41 (18)	H11B—C11—H11C	109.5
C3—C2—H2	120.3	N3—C12—C13	113.92 (16)
C1—C2—H2	120.3	N3—C12—H12A	108.8
C2—C3—C4	120.08 (19)	C13—C12—H12A	108.8
C2—C3—H3	120.0	N3—C12—H12B	108.8
C4—C3—H3	120.0	C13—C12—H12B	108.8
C3—C4—C5	119.69 (19)	H12A—C12—H12B	107.7
C3—C4—H4	120.2	C12—C13—H13A	109.5
C5—C4—H4	120.2	C12—C13—H13B	109.5
C4—C5—C6	121.87 (17)	H13A—C13—H13B	109.5
C4—C5—H5A	119.1	C12—C13—H13C	109.5
C6—C5—H5A	119.1	H13A—C13—H13C	109.5
C5—C6—C1	116.39 (17)	H13B—C13—H13C	109.5
C5—C6—C7	121.92 (15)	N3—C14—C15	112.69 (16)
C1—C6—C7	121.34 (16)	N3—C14—H14A	109.1
O1—C7—N1	122.84 (17)	C15—C14—H14A	109.1
O1—C7—C6	121.22 (15)	N3—C14—H14B	109.1
N1—C7—C6	115.87 (15)	C15—C14—H14B	109.1
N2—C8—C9	113.5 (2)	H14A—C14—H14B	107.8
N2—C8—H8A	108.9	C14—C15—H15A	109.5
C9—C8—H8A	108.9	C14—C15—H15B	109.5
N2—C8—H8B	108.9	H15A—C15—H15B	109.5
C9—C8—H8B	108.9	C14—C15—H15C	109.5
H8A—C8—H8B	107.7	H15A—C15—H15C	109.5
C9A—C8A—N2	106.9 (6)	H15B—C15—H15C	109.5
C9A—C8A—H8AA	110.3		
			
O2—P1—N1—C7	158.75 (14)	C4—C5—C6—C7	171.39 (17)
N2—P1—N1—C7	−75.93 (16)	C2—C1—C6—C5	0.7 (3)
N3—P1—N1—C7	38.97 (17)	C2—C1—C6—C7	−172.59 (17)
O2—P1—N2—C8	−72.0 (2)	P1—N1—C7—O1	17.5 (3)
N3—P1—N2—C8	51.5 (2)	P1—N1—C7—C6	−159.56 (12)
N1—P1—N2—C8	170.05 (18)	C5—C6—C7—O1	−139.50 (18)
O2—P1—N2—C10	97.97 (16)	C1—C6—C7—O1	33.4 (3)
N3—P1—N2—C10	−138.49 (15)	C5—C6—C7—N1	37.6 (2)
N1—P1—N2—C10	−19.96 (17)	C1—C6—C7—N1	−149.49 (17)
O2—P1—N2—C8A	−37.8 (5)	C10—N2—C8—C9	−56.1 (3)
N3—P1—N2—C8A	85.7 (5)	C8A—N2—C8—C9	37.9 (6)
N1—P1—N2—C8A	−155.7 (5)	P1—N2—C8—C9	114.5 (2)
O2—P1—N3—C14	159.56 (14)	C8—N2—C8A—C9A	−28.3 (5)
N2—P1—N3—C14	31.58 (16)	C10—N2—C8A—C9A	74.1 (8)
N1—P1—N3—C14	−83.15 (16)	P1—N2—C8A—C9A	−146.0 (6)
O2—P1—N3—C12	−39.48 (15)	C8—N2—C10—C11	100.8 (2)
N2—P1—N3—C12	−167.46 (13)	C8A—N2—C10—C11	63.7 (5)
N1—P1—N3—C12	77.81 (15)	P1—N2—C10—C11	−69.5 (2)
C6—C1—C2—C3	0.7 (3)	C14—N3—C12—C13	77.6 (2)
C1—C2—C3—C4	−1.0 (3)	P1—N3—C12—C13	−85.37 (18)
C2—C3—C4—C5	−0.1 (3)	C12—N3—C14—C15	75.8 (2)
C3—C4—C5—C6	1.6 (3)	P1—N3—C14—C15	−122.62 (16)
C4—C5—C6—C1	−1.9 (3)		

### Hydrogen-bond geometry (Å, °)

**Table:**

*D*—H···*A*	*D*—H	H···*A*	*D*···*A*	*D*—H···*A*
N1—H1N···O2^i^	0.86 (2)	1.93 (2)	2.7714 (19)	167 (2)

Symmetry codes: (i) −*x*+1, −*y*+1, −*z*+1.
